# Job titles classified into socioeconomic and occupational groups identify subjects with increased risk for respiratory symptoms independent of occupational exposure to vapour, gas, dust, or fumes

**DOI:** 10.1080/20018525.2018.1468715

**Published:** 2018-05-15

**Authors:** Christian Schyllert, Martin Andersson, Linnea Hedman, Magnus Ekström, Helena Backman, Anne Lindberg, Eva Rönmark

**Affiliations:** a Department of Public Health and Clinical Medicine, Division of Occupational and Environmental Medicine, The OLIN unit, Umeå University, Umeå, Sweden; b Department of Health Sciences, Division of Nursing, Luleå University of Technology, Luleå, Sweden; c Department of Respiratory Medicine and Allergology, Institution for Clinical Sciences, Lund University, Lund, Sweden; d Department of Public Health and Clinical Medicine, Division of Medicine, Umeå University, Umeå, Sweden

**Keywords:** Asthma, occupational exposure, socioeconomic status

## Abstract

**Objectives**: To evaluate the ability of three different job title classification systems to identify subjects at risk for respiratory symptoms and asthma by also taking the effect of exposure to vapours, gas, dust, and fumes (VGDF) into account.

**Background**: Respiratory symptoms and asthma may be caused by occupational factors. There are different ways to classify occupational exposure. In this study, self-reported occupational exposure to vapours, gas, dust and fumes was used as well as job titles classifed into occupational and socioeconomic Groups according to three different systems.

**Design:** This was a large population-based study of adults aged 30–69 years in Northern Sweden (*n* = 9,992, 50% women). Information on job titles, VGDF-exposure, smoking habits, asthma and respiratory symptoms was collected by a postal survey. Job titles were used for classification into socioeconomic and occupational groups based on three classification systems; Socioeconomic classification (SEI), the Nordic Occupations Classification 1983 (NYK), and the Swedish Standard Classification of Occupations 2012 (SSYK). Associations were analysed by multivariable logistic regression.

**Results**: Occupational exposure to VGDF was a risk factor for all respiratory symptoms and asthma (odds ratios (ORs) 1.3–2.4). Productive cough was associated with the socioeconomic groups of manual workers (ORs 1.5–2.1) and non-manual employees (ORs 1.6–1.9). These groups include occupations such as construction and transportation workers, service workers, nurses, teachers and administration clerks which by the SSYK classification were associated with productive cough (ORs 2.4–3.7). Recurrent wheeze was significantly associated with the SEI group manual workers (ORs 1.5–1.7). After adjustment for also VGDF, productive cough remained significantly associated with the SEI groups manual workers in service and non-manual employees, and the SSYK-occupational groups administration, service, and elementary occupations.

**Conclusions**: In this cross-sectional study, two of the three different classification systems, SSYK and SEI gave similar results and identified groups with increased risk for respiratory symptoms while NYK did not give conclusive results. Furthermore, several associations were independent of exposure to VGDF indicating that also other job-related factors than VGDF are of importance.

## Introduction

It is well established that respiratory symptoms and asthma may be caused by occupational factors [1]. Occupational exposures have been related to adult-onset asthma and work-aggravated asthma, and the population-attributable fraction is estimated at about 15% [2]. Several jobs and occupational exposures are strongly associated with cough, wheeze, and nasal symptoms [3–6], and low socioeconomic status is associated with asthma, cough, and wheeze [7]. In population-based studies, occupational exposure to vapours, gas, dust, and fumes (VGDF) have been shown to increase the risk of asthma [8], respiratory symptoms [9] and rhinorrhoea [10].

There are different methods to classify occupational exposure. Most studies in occupational epidemiology are based on specific workforces or exposure to specific agents [3,6,11–13]. In large population-based studies, occupational exposures are commonly assessed by using a job-exposure matrix (JEM) based on job title, or by using a single-item question of exposures to VGDF. There is no consensus whether the use of a JEM or the use of a VGDF question is the best way of assessing occupational exposure in epidemiological studies [14–16]. While job titles are frequently used to construct a JEM or for classifying socioeconomic status [7], job titles grouped into occupational groups have seldom been analysed as risk factors for respiratory illness.

Throughout the Nordic countries, similar systems based on job titles, are used to classify different occupational groups. The International Standard Classification of Occupations (ISCO) 08 [17] is used as a basis for the current standards of occupation classification in Sweden (SSYK) [18], Norway (STYRK) [19], Denmark (DISCO) [20], and Finland (Ammattiluokitus) [21]. The Icelandic standard (Istarf) [22] is based on a previous, but similar, issue: ISCO 88. The Nordic Classification of Occupations (NYK) [23] is based on ISCO 68, an older system of which the last version was published in 1983 and has since been replaced by SSYK. For socioeconomic status, different classifications systems are used in the Nordic countries. To the best of our knowledge, no previous study has simultaneously evaluated the associations between respiratory health and occupation, socioeconomic status, and occupational exposure in the same population.

In this study, we have used job titles for classification of occupational groups and socioeconomic groups by three different classification systems, and analysed the associations with respiratory symptoms and asthma by also taking exposure to VGDF into account. We hypothesised that the three systems would yield similar results.

## Methods

### Study population

This was a large population-based cross-sectional study performed within the epidemiological research program the Obstructive Lung disease In Northern Sweden (OLIN) studies. In 2006, a random sample of the population aged 20–69 years in the county of Norrbotten was invited to a postal questionnaire survey (*n* = 7,997) and 77% participated. A cohort recruited to a similar survey in 1996 was at the same time in 2006 invited for follow-up, now aged 30–84 years (*n* = 7,004) and 85% participated. In total 12,055 (80.4%) responded and the same questionnaire was used in both cohorts [24]. The questionnaire was based on a British Medical Research Council questionnaire but included questions from the US Tucson and ATS questionnaires and has been described previously [25] The study population in the current study includes all individuals 30–69 years of age (*n* = 9,992, 50.3% women). The younger and older individuals were excluded since a large portion of them were not professionally active. Of those below 30 years of age, 45% were students or did not report any occupation. The study was approved by the Regional Ethical Review Board of Umeå, Sweden.

### Definitions

Longest held job title was used to classify occupational groups by two different systems: (1) **the Nordic Occupations Classification 1983 (NYK)** [23]: Science, humanistic, and artistic work (*Science*), Health and social work (*Healthcare*), Administration, clerical, and commercial work (*Administration*), Agriculture, forestry, and fishery (*Agriculture*), Mining, quarrying, and petroleum extraction work (*Mining*), Transportation and communication work (*Transportation*), Manufacturing and machine maintenance (*Manufacturing*) Service work (*Service*), Other and unspecified (*Other*) including students, professional military personnel and non-classifiable individuals; (2) **the Swedish Standard Classification of Occupations 2012 (SSYK)** [18]: Occupations requiring advanced level of higher education (*Occupations requiring advanced education*), Occupations requiring higher education qualifications or equivalent (*Occupations requiring higher education*), Administration and customer service clerks (*Administration*), Service, care, and shops sales workers (*Service*), Agricultural horticultural, forestry, and fishery work (*Agriculture*), Building and manufacturing work (*Building*), Mechanical manufacturing and transport work (*Manufacturing*), Elementary occupations (*Elementary*), and Other and unspecified with students, professional military personnel and non-classifiable individuals (*Other*). Longest held job title was also used to classify socioeconomic status groups according to the Swedish **Socioeconomic classification (SEI)** [26]: Manual workers in industry (*Manual work industry*), Manual workers in service (*Manual work service*), Non-manual employees, lower level (*Non-manual employees L*) and intermediate level (*Non-manual employees I*), Professionals and executives (*Professionals and executives)*, Self-employed non-professionals (*Self-employed non-prof*), and Other and unspecified (*Other*) with students and non-classifiable individuals. All three classification systems are issued by Statistics Sweden and based on ISCO. Exposure to vapour, gas, dust or fumes (VGDF) was based on the question: Have you, in your work, been heavily exposed to vapours, gas, dust, and/or fumes? Of the 9,992 subjects, 2.3% did not answer this question.

**Productive cough**: Do you usually have phlegm when coughing, or do you have phlegm in your chest which is difficult to bring up, and have you had this during most days for at least three months? **Recurrent wheeze**: Do you usually have wheeze, whistling, or a noisy sound in your chest when breathing? **Allergic rhino-conjunctivitis**: Do you have allergic rhinitis (hay-fever) or allergic eye symptoms? **Use of asthma medication**: Do you currently use asthma medicines (regularly or as needed)? **Physician diagnosis of asthma**: Have you been diagnosed as having asthma by a physician? **Current asthma**: Affirmative answer to physician diagnosis of asthma and either recurrent wheeze or use of asthma medication during the last 12 months. **Allergic asthma**: Affirmative answers to current asthma and allergic rhino-conjunctivitis. **Non-allergic asthma**: Affirmative answer to current asthma and negative answer to allergic rhino-conjunctivitis. **Age groups**: age grouped into 30–39, 40–49, 50–59, and 60–69 years of age. **Smoking habits**: non-smoker, ex-smoker (stopped more than 12 months ago) and current smoker. **Family history of asthma**: Have any of your parents or siblings asthma, or have they had asthma?

### Statistics

Pearson’s *χ* ^2^ test was used for comparing prevalence between groups. Multivariable logistic regression models were used to calculate odds ratios (OR) and 95% confidence intervals (95% CI) of respiratory symptoms and asthma in relation to self-reported VGDF-exposure, SEI and occupational groups (NYK and SSYK), respectively. For SEI and SSYK *professionals and executives*, and *managers*, respectively, were used as reference groups. For NYK, the largest group, *administration*, was used as reference group. The logistic regression models were adjusted for sex, age, family history of asthma, and smoking habits. Separate models were additionally adjusted for VGDF-exposure. Furthermore, for the variables productive cough, recurrent wheeze, and current asthma, the logistic regression analyses were performed stratified by quartiles of the number of years of working in the main occupation. The logistic regression analyses were also performed after excluding subjects with onset of asthma before the age of 18 years and are presented in online table 2. The logistic regression analyses of the confounding factors are presented in online table 3. Statistical significance was defined as a two-sided *p* < 0.05. All analyses were carried out using IBM SPSS Statistics version 23 (IBM Corp, New York, NY, USA).

## Results

### Basic characteristics

Subjects in ages 40–59 years reported the highest prevalence of smoking. Productive cough was most prevalent in the oldest age groups, while current asthma, allergic rhino-conjunctivitis and rhinitis were most prevalent in the younger age groups. The prevalence of current asthma, allergic rhino-conjunctivitis, and rhinitis were higher among women than men (Table 1). The largest SEI groups were *manual workers service* and *industry*, the former dominated by women and the latter by men. The largest occupational NYK-groups were *administration* and *manufacturing*. The largest SSYK-groups were *service* and *occupations requiring advanced education*. Of the total population, 30.9% reported exposure to VGDF with the highest prevalence among older subjects and men (Table 2).

**Table 1.** Prevalence (%) of family history of asthma, smoking habits, respiratory symptoms, and asthma in the population, by age groups and by sex.

		Age groups					Sex		
	All	30–39 y	40–49 y	50–59 y	60–69 y	*p χ*^2*^	Women	Men	*p χ*^2**^
	(*n* = 9,992)	(*n* = 2,050)	(*n* = 2,455)	(*n* = 2,868)	(*n* = 2,619)		(*n* = 5,030)	(*n* = 4,962)	
Family history of asthma	22.0	24.5	24.7	22.2	17.4	**<0.001**	25.0	19.0	**<0.001**
Smoking habits									
Non-smoker	54.7	73.7	57.9	45.6	46.6		52.9	56.5	
Ex-smoker	25.7	13.6	20.4	30.3	35.0	**<0.001**	24.4	26.9	**<0.001**
Current smoker	19.2	12.4	21.0	23.7	17.8		22.3	16.0	
Respiratory symptoms and asthma									
Productive cough	9.1	6.0	7.9	9.7	12.0	**<0.001**	9.0	9.2	0.697
Recurrent wheeze	12.1	11.3	12.5	12.6	11.8	0.485	12.1	12.1	0.970
Allergic rhino-conjunctivitis	23.9	31.4	28.3	22.3	15.5	**<0.001**	25.6	22.1	**<0.001**
Rhinitis	21.6	23.8	22.5	21.3	19.4	**0.002**	22.8	20.4	**0.003**
Current asthma	9.2	9.8	10.3	8.5	8.4	**0.048**	10.1	8.3	**0.002**
Allergic asthma	5.4	6.9	6.9	5.1	3.1	**<0.001**	6.0	4.8	**0.011**
Non-allergic asthma	3.8	2.9	3.3	3.5	5.3	**<0.001**	4.1	3.4	0.080

*comparing age-groups. **comparing sex. ***comparing all SSYK/NYK/SEI groups respectively. ****comparing VGDF to No VGDF.

**Table 2.** Prevalence (%) of socioeconomic groups (SEI), occupational groups (NYK and SSYK), and self-reported occupational exposure to VGDF in the population, by age groups and by sex.

		Age groups				Sex	
	All	30–39 y	40–49 y	50–59 y	60–69 y	Women	Men
	(*n* = 9,992)	(*n* = 2,050)	(*n* = 2,455)	(*n* = 2,868)	(*n* = 2,619)	(*n* = 5,030)	(*n* = 4,962)
SEI							
Manual workers industry	21.8	18.0	20.9	24.0	23.2	3.5	40.3
Manual workers service	27.5	26.0	29.9	26.0	27.9	39.2	15.6
Non-manual employees L	14.2	10.5	13.3	16.0	15.8	18.7	9.6
Non-manual employees I	20.6	23.5	20.3	20.3	19.0	24.3	16.9
Professionals and exec	6.3	8.0	6.8	6.2	4.7	4.6	8.1
Self-employed non-prof	2.4	1.4	2.1	2.6	3.1	1.4	3.4
Other	7.3	12.6	6.8	4.9	6.3	8.4	6.2
NYK							
Science	13.1	13.9	13.2	12.6	13.1	15.4	10.8
Healthcare	18.3	18.0	20.8	20.3	14.1	32.4	4.1
Administration	20.0	19.5	18.8	19.5	22.0	24.7	15.1
Agriculture	2.9	2.0	2.9	2.7	3.9	1.1	4.7
Mining	1.5	0.7	1.1	1.8	2.1	0.1	2.8
Transportation	5.0	4.2	5.1	4.7	6.0	2.2	7.9
Manufacturing	20.0	18.6	20.4	21.8	18.8	3.6	36.6
Service	7.9	8.3	7.7	6.5	9.1	12.0	3.7
Other	11.3	14.7	10.2	10.2	10.9	8.4	14.2
SSYK							
Managers	1.7	1.3	1.9	2.2	1.5	1.3	2.2
Occupations requiring advanced education	17.0	20.9	17.2	16.5	14.3	22.8	11.1
Occupations requiring higher education	8.0	10.4	7.5	7.6	7.2	6.5	9.6
Administration	10.4	6.4	9.8	11.8	12.6	15.3	5.5
Service	20.8	21.0	23.1	20.2	19.3	33.5	8.0
Agriculture	3.0	2.2	3.0	2.7	3.8	1.2	4.8
Building	13.9	13.8	13.9	15.1	12.7	1.4	26.6
Manufacturing	10.0	7.3	9.3	10.5	12.2	2.4	17.7
Elementary	5.0	4.2	5.3	4.3	6.1	8.4	1.5
Other	10.1	12.5	9.0	9.1	10.4	7.3	13.0
Occupational exposure							
VGDF	30.9	25.6	29.1	32.8	34.5	14.8	47.2

*comparing age-groups. **comparing sex . ***comparing all SSYK/NYK/SEI groups respectively. ****comparing VGDF to No VGDF.

### Prevalence of respiratory symptoms in relation to socioeconomic group and occupational groups

According to SEI, current asthma and all respiratory symptoms were most prevalent among *manual workers industry* and *service*. Within the NYK-occupational groups, productive cough and recurrent wheeze were most prevalent among *mining, manufacturing,* and *service* while current asthma was most prevalent among *healthcare* and *service*. Regarding the SSYK-occupational groups, productive cough was most prevalent among *administration, agriculture, manufacturing,* and *elementary*, while recurrent wheeze was most prevalent among *service, building, manufacturing,* and *elementary*. All respiratory symptoms and asthma were significantly more prevalent among subjects reporting exposure to VGDF compared to non-exposed (Table 3).

**Table 3.** Prevalence (%) of respiratory symptoms and asthma by socioeconomic groups (SEI), occupational groups (NYK and SSYK), and self-reported occupational exposure to VGDF.

.	Productive cough	Recurrent wheeze	Allergic rhino-conjunctivitis	Rhinitis	Current asthma	Allergic asthma	Non-allergic asthma
All	9.1	12.1	23.9	21.6	9.2	5.4%	3.8%
SEI							
Manual workers industry	10.8	14.6	20.9	22.1	9.0	4.8	4.1
Manual workers service	10.1	14.1	24.5	24.3	11.0	6.3	4.7
Non-manual employees L	9.5	10.8	23.8	21.4	7.9	4.3	3.5
Non-manual employees I	7.4	9.1	25.9	18.9	8.7	6.0	2.7
Professionals and exec	4.7	8.2	27.2	20.1	8.4	4.7	3.6
Self-employed non-prof	7.2	10.2	16.2	17.0	6.8	3.4	3.4
*p χ*^2***^	**<0.001**	**<0.001**	**<0.001**	**<0.001**	**0.008**	0.053	**0.013**
NYK							
Science	7.5	9.2	25.7	21.6	8.9	6.0	2.9
Healthcare	8.1	12.7	25.6	22.5	11.0	6.2	4.8
Administration	9.2	10.7	25.4	21.3	8.4	5.2	3.2
Agriculture	10.6	11.6	20.2	18.5	9.2	4.8	4.5
Mining	13.1	15.9	18.6	21.4	4.8	2.1	2.8
Transportation	9.1	13.3	22.4	23.2	8.3	5.8	2.6
Manufacturing	10.2	14.8	21.7	21.7	9.3	5.2	4.1
Service	10.7	13.2	22.9	24.2	10.2	5.1	5.1
*p χ*^2***^	**0.041**	**<0.001**	**0.014**	0.157	**0.042**	0.364	**0.024**
SSYK							
Managers	4.0	7.5	23.6	23.6	5.2	3.4	1.7
Occupations req. advanced education	6.8	8.8	26.8	20.1	9.1	5.9	3.2
Occupations req. higher education	7.9	9.0	25.8	19.1	8.6	5.9	2.7
Administration	10.0	10.6	24.5	22.0	8.0	4.4	3.6
Service	9.1	13.5	25.0	23.9	10.6	6.1	4.5
Agriculture	10.5	12.2	20.3	19.3	9.8	5.1	4.7
Building	9.4	13.8	21.5	22.7	9.0	5.0	4.0
Manufacturing	11.2	16.3	20.6	20.7	8.9	5.0	3.9
Elementary	14.1	16.8	24.4	26.5	10.9	5.7	5.3
*p χ*^2***^	**<0.001**	**<0.001**	**0.002**	**0.001**	0.118	0.557	0.125
Occupational exposure							
No VGDF	7.0	9.2	23.3	19.1	7.7	4.6	3.1
VGDF	13.7	18.6	25.3	27.2	12.4	7.1	5.3
*p χ*^2****^	**<0.001**	**<0.001**	**0.031**	**<0.001**	**<0.001**	**<0.001**	**<0.001**

*comparing age-groups. **comparing sex. ***comparing all SSYK/NYK/SEI groups respectively. ****comparing VGDF to No VGDF.

### Socioeconomic group as a risk factor for respiratory symptoms and asthma

In logistic regression models adjusted for sex, age, family history of asthma, and smoking habits, *manual workers industry,* and *service* were significantly associated with productive cough and recurrent wheeze. *Non-manual employees* had increased risk of productive cough (Table 4). When adjusted also for VGDF-exposure, *manual workers service* and *non-manual employees* remained significantly associated with productive cough (Table 4). When stratifying for years in occupation, the risk for productive cough was increased in the 1st and 2nd quartiles among *manual workers*, while the risk for recurrent wheeze and current asthma was increased in the 3rd quartile among *manual workers* and *non-manual employees* (online table 1).

**Table 4.** Risk for respiratory symptoms and asthma by socioeconomic group (SEI), occupational groups (NYK and SSYK), and self-reported occupational exposure to VGDF analysed by multivariable logistic regression and expressed as odds ratios (OR) and 95% confidence intervals (95% CI). All analyses were adjusted for sex, age, family history of asthma and smoking habits. Significant results in bold, borderline significant results in bold italic.

	Productive cough			Recurrent wheeze			Allergic rhino-conjunctivitis			Rhinitis			Current asthma			Allergic asthma			Non-allergic asthma		
	OR	95% CI		OR	95% CI		OR	95% CI		OR	95% CI		OR	95% CI		OR	95% CI		OR	95% CI	
SEI																					
Manual workers industry	**2.08**	**1.40**	**3.10**	**1.70**	**1.24**	**2.34**	0.81	0.65	1.00	1.15	0.92	1.44	1.13	0.81	1.57	1.15	0.75	1.77	1.07	0.67	1.73
Manual workers service	**1.93**^a^	**1.30**	**2.86**	**1.54**	**1.13**	**2.11**	0.87	0.71	1.07	1.14	0.92	1.42	1.21	0.88	1.65	1.29	0.86	1.94	1.07	0.67	1.70
Non-manual employees L	**1.85**^a^	**1.22**	**2.79**	1.22	0.87	1.71	0.88	0.70	1.10	1.03	0.81	1.30	0.84	0.59	1.20	0.91	0.57	1.43	0.79	0.47	1.31
Non-manual employees I	**1.57**^a^	**1.05**	**2.36**	1.11	0.80	1.55	0.93	0.76	1.14	0.90	0.72	1.14	0.99	0.71	1.37	1.27	0.84	1.93	0.67	0.40	1.10
Professionals and exec	1			1			1			1			1			1			1		
Self-employed non-prof	1.35	0.72	2.51	1.21	0.72	2.04	0.60	0.40	0.88	0.85	0.57	1.26	0.82	0.45	1.47	0.81	0.36	1.80	0.83	0.36	1.89
NYK																					
Science	0.85	0.65	1.10	0.88	0.69	1.12	0.97	0.83	1.15	1.03	0.87	1.22	1.05	0.82	1.35	1.13	0.83	1.53	0.91	0.61	1.38
Healthcare	0.87	0.69	1.10	1.13	0.92	1.39	0.93	0.80	1.08	0.99	0.84	1.16	***1.25***	***1.00***	***1.56***	1.08	0.81	1.43	**1.50**^a^	**1.07**	**2.11**
Administration	1			1			1			1			1			1			1		
Agriculture	1.11	0.73	1.68	1.04	0.69	1.56	0.81	0.59	1.10	0.90	0.65	1.24	1.16	0.75	1.80	0.99	0.55	1.78	1.38	0.74	2.59
Mining	1.29	0.76	2.19	1.39	0.84	2.28	0.79	0.51	1.23	1.08	0.71	1.65	0.56	0.25	1.24	0.44	0.13	1.41	0.75	0.26	2.13
Transportation	0.92	0.65	1.31	1.19	0.87	1.62	0.91	0.72	1.16	1.14	0.90	1.45	1.02	0.71	1.48	1.19	0.77	1.85	0.78	0.42	1.46
Manufacturing	1.11	0.88	1.41	**1.41**	**1.14**	**1.74**	0.88	0.74	1.03	1.08	0.91	1.27	1.20	0.94	1.53	1.11	0.82	1.51	1.30	0.90	1.88
Service	1.10	0.83	1.45	1.13	0.87	1.46	0.86	0.71	1.05	1.11	0.91	1.36	1.20	0.90	1.60	0.94	0.64	1.38	**1.57**	**1.05**	**2.37**
SSYK																					
Managers	1			1			1			1			1			1			1		
Occupations req. advanced education	1.99	0.91	4.38	1.30	0.71	2.37	1.06	0.73	1.54	0.79	0.55	1.15	1.75	0.87	3.53	1.56	0.67	3.66	1.94	0.60	6.32
Occupations req. higher education	2.15	0.96	4.80	1.23	0.66	2.30	1.03	0.70	1.53	0.75	0.50	1.11	1.69	0.82	3.48	1.61	0.67	3.85	1.69	0.50	5.74
Administration	**2.61^a^**	**1.19**	**5.76**	1.41	0.77	2.60	1.04	0.71	1.54	0.87	0.59	1.28	1.47	0.72	3.02	1.21	0.51	2.92	1.92	0.58	6.35
Service	**2.35^a^**	**1.08**	**5.13**	1.76	0.98	3.19	0.98	0.67	1.42	0.91	0.63	1.32	1.94	0.97	3.89	1.56	0.67	3.62	2.52	0.78	8.12
Agriculture	**2.63**	**1.13**	**6.17**	1.64	0.83	3.23	0.84	0.53	1.33	0.79	0.50	1.25	2.04	0.93	4.46	1.53	0.58	4.05	2.82	0.79	10.04
Building	**2.41**	**1.10**	**5.28**	**1.96**	**1.08**	**3.56**	0.90	0.62	1.32	0.96	0.66	1.40	1.96	0.97	3.98	1.59	0.67	3.77	2.50	0.77	8.17
Manufacturing	**2.69**	**1.22**	**5.92**	**2.21**	**1.21**	**4.03**	0.88	0.59	1.30	0.84	0.57	1.23	1.87	0.91	3.82	1.55	0.65	3.71	2.32	0.70	7.65
Elementary	**3.66**^a^	**1.63**	**8.21**	**2.13**	**1.14**	**3.99**	1.03	0.68	1.56	1.06	0.70	1.60	2.02	0.96	4.24	1.53	0.61	3.80	2.78	0.82	9.44
Occupational exposure																					
No VGDF	1			1			1			1			1			1			1		
VGDF	**2.10**	**1.80**	**2.45**	**2.36**	**2.06**	**2.71**	**1.32**	**1.18**	**1.47**	**1.76**	**1.58**	**1.97**	**2.01**	**1.72**	**2.35**	**1.96**	**1.60**	**2.38**	**1.87**	**1.48**	**2.36**
Occupational exposure (among non-smokers*)																					
No VGDF	1			1			1			1			1			1			1		
VGDF	**2.21**	**1.76**	**2.78**	**2.46**	**2.00**	**3.03**	**1.30**	**1.13**	**1.51**	**1.83**	**1.57**	**2.14**	**1.95**	**1.57**	**2.43**	**1.88**	**1.45**	**2.45**	**1.87**	**1.32**	**2.66**

^a^ Significantly increased when also adjusted for VGDF. *The analyses were performed among non-smokers only.

### Occupational group as a risk factor for respiratory symptoms and asthma

Adjusted analyses were also performed for the occupational groups. Regarding the NYK-occupational groups, *healthcare* and *service* were both significantly associated with non-allergic asthma while *manufacturing* was associated with recurrent wheeze (Table 4). When stratifying for years in occupation, *transportation* in the 3rd quartile had increased risk of current asthma, and the 3rd and 4th quartiles of *manufacturing* had increased risk of recurrent wheeze (online table 1).

For the SSYK-occupational groups, all groups but *occupations requiring advanced* or *higher education* were associated with productive cough. *Building, manufacturing,* and *elementary* were also associated with recurrent wheeze. The association between *administration, service,* and *elementary* and productive cough remained statistically significant when also adjusting for occupational exposure to VGDF.

### Occupational exposure to VGDF as risk factor for respiratory symptoms and asthma

Occupational exposure to VGDF was associated with increased risks of all outcomes. Analyses executed among only non-smokers yielded similar results (Table 4). When stratifying for years in occupation, the risks remained significantly increased in all quartiles (online table 1).

### Prevalence of occupational exposure to VGDF in the different socioeconomic and occupational groups

**Figure 1.**
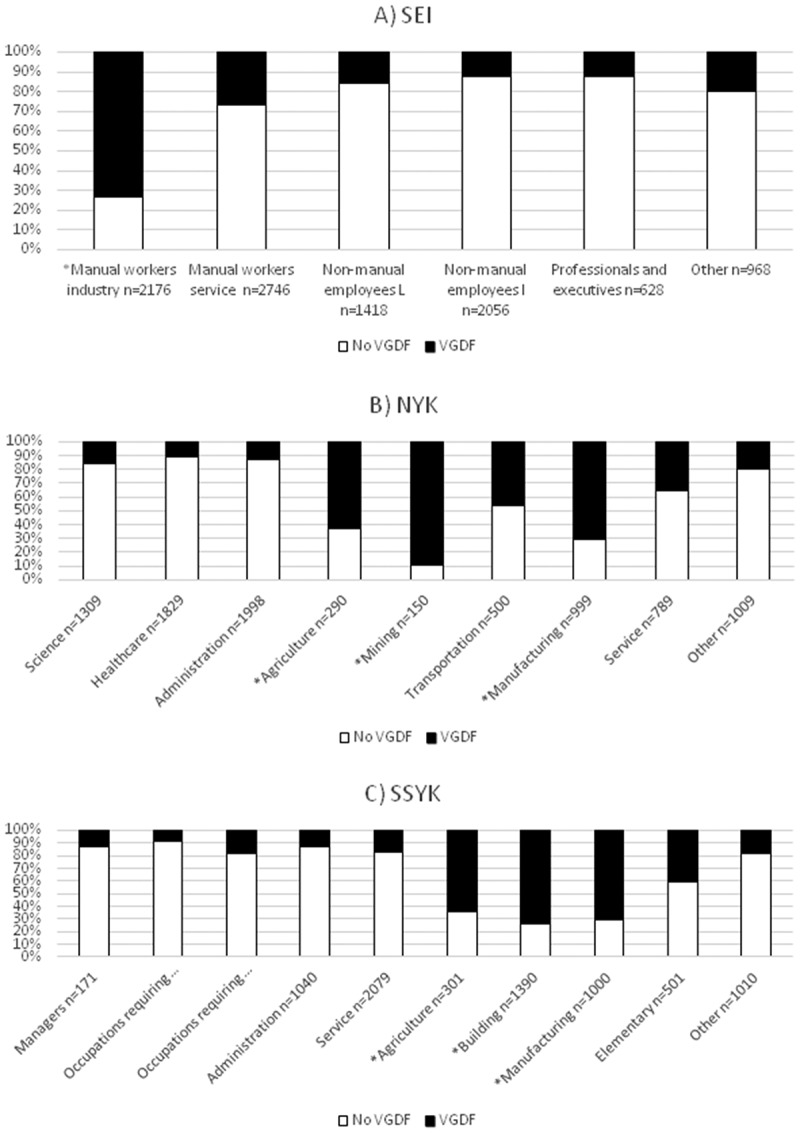
Proportion (%) reporting exposure to VGDF in the socioeconomic (A) and occupational (B, C) groups. * = a significant difference in proportion reporting VGDF in the specific group compared to all other groups.

Within the SEI groups, *manual workers in industry* reported significantly higher prevalence of occupational exposure to VGDF compared to the rest of the population (Figure 1(A)). In the NYK-occupational groups this was true for *agriculture, mining,* and *manufacturing* (Figure 1(B)), and for the SSYK-occupational groups: *agriculture, building,* and *manufacturing* (Figure 1(C)).

### Sensitivity analysis

When excluding individuals with asthma onset before the age of 18 years, the results did not change significantly (online table 2).

## Discussion

In this population-based study, we found that two of the systems of classifying job titles, SEI and SSYK, gave in most aspects similar results and could identify subjects with increased risk for respiratory symptoms also after adjusting for exposure to VGDF, while the third, NYK, did not give any conclusive results. We found associations between productive cough and manual workers as well as non-manual employees. Within these socioeconomic groups, there are jobs such as builders, transportation workers, service workers, nurses, teachers, and administration clerks which also were found in the SSYK-groups associated with respiratory symptoms. Not surprisingly, the reported prevalence of exposure to VGDF was highest among subjects belonging to these occupational and socioeconomic risk groups. However, the association between respiratory symptoms and these socioeconomic and occupational groups remained after adjustment for exposure to VGDF, indicating that other factors than VGDF may contribute to the observed associations.

A Swedish study from the mid-eighties and early nineties reported associations between wheeze and cough, and miners [27], as well as the SEI groups of manual workers and self-employed non-professionals [25]. The former corresponds well with the findings in our study. In the latter study [25], asthma was associated with workers in trade and commerce as well as healthcare and administration, while in the current study we only found an association between healthcare and asthma. These differences could mirror the changes in working conditions between the late twentieth and early twenty-first centuries, but also changes in smoking habits and living conditions [28]. Our finding of an association between healthcare and asthma corresponds with a study from Finland published in 2001 [29] that showed associations between incident asthma and medical and nursing work. They also found associations between incident asthma and jobs in the sectors of sales, agriculture, mining, transportation, manufacturing and service work. In our study, these sectors were associated with cough and wheeze, but not asthma. In line with our findings, another Swedish study published in 2006 [7] showed that manual workers had an increased incidence rate of shortness of breath, wheeze, asthma, and cough. Interestingly, both the Finnish [29] and latter Swedish [7] studies had a longitudinal design, while the present study was cross-sectional which could affect the results; the main findings were similar.

The NYK classification system was the least sensitive for identifying occupational groups associated with respiratory symptoms. Thus our hypothesis was not verified. The latest version of the NYK-manual is from 1983 and has been replaced with SSYK, of which the latest version came in 2012. A possible explanation for the weak associations between NYK and respiratory symptoms could be that NYK is an older way of classifying occupations, not reflecting modern working life and working conditions. Furthermore, in NYK there is no obvious occupational category to use as reference in the adjusted analyses. In both SSYK and SEI, we used the managers, professionals, and executives as reference, while in NYK we used the administration group as reference as this was the largest group. This could skew the results somewhat; the occupations within this group can be found not only among the SEI group of professionals and executives, but also among non-manual employees, who had an increased risk of some of the respiratory symptoms.

It has previously been shown that exposure to VGDF is associated with cough and wheeze as well as both asthma and rhinitis [4,8–10]. Also our findings support this; VGDF-exposure was significantly associated to all symptoms and diseases, and the results were almost identical when the analyses were performed in non-smokers only. All models including occupational and SEI groups were also performed adjusted for VGDF-exposure. In these analyses, most of the identified associations between respiratory symptoms and diseases and SEI and SSYK remained. This indicates that other factors than smoking and occupational exposure to VGDF contributes to the risk of respiratory symptoms associated with socioeconomic and occupational groups. One possible explanation could be that SEI is associated with lifestyle factors such as BMI, physical activity, or living conditions [30]. Unfortunately, apart from smoking habits, we were lacking such information in our study. Another possibility could be that while a question of being exposed to VGDF is very inclusive, it might still miss some exposures that are more common in some lines of work, e.g. occupations that might not be considered dirty. In these occupational groups, we find typically female-dominated occupations such as nurses, cleaners, hairdresser, social workers and administration clerks among others. This assumption is supported by a recent US study [31] where the highest prevalence of asthma was found among the occupations in office and administrative support, healthcare practitioners and sales.

In occupational epidemiology, the healthy worker effect must be considered [32–34]. When the analyses of socioeconomic and occupational groups were stratified for number of years in main occupation, the associations were in general statistically significant for those who had worked 21–30 years, but not for those who had worked more than 30 years. This could be a healthy worker effect; the workers that stay the longest are respiratory healthier, not displaying symptoms. Furthermore, some of those who reported the highest number of working years might have retired when the data collection was conducted, and their respiratory symptoms, if any, may have since remitted. Due to the cross-sectional design of the study, and the fact that we lack data on occupation before onset of respiratory symptoms, we cannot draw any conclusions on causality. Nevertheless, we found similar results when subjects with onset of asthma before the age of 18 were excluded. In the current study, we have used ‘longest held job’ which can create bias as exposure to specific agents in certain occupations can cause symptoms after a shorter period of time, and we do not have data on job changes. However, our results indicate that symptoms such as cough may develop after a shorter period of exposure while a longer period of exposure is needed for symptoms such as wheeze. Further, we have in a previous study found indications that different types of exposure associate with different types of respiratory symptoms [35].

The job titles and occupational exposure to VGDF were self-reported, and recall bias cannot be ruled out; those with respiratory symptoms/asthma may have been more prone to report previous occupational exposure. Occupational exposure can be estimated using a single-item question of exposure or by using a JEM based on job titles [14,36]. Some argue that a JEM leads to more accurate measures of occupational exposure, while others have reported that the difference between self-reporting and JEM are less than expected and that a question of VGDF-exposure is applicable in epidemiological research [14–16]. However, it should be noted that self-reporting usually yields higher risks and more strongly statistically significant results than a JEM [14]. The strength with our study was the large population-based cohort with a high participation rate resulting in a representative study population. Furthermore, the questionnaire and questions [7–10,14,16,24,26,28,35] have been used in several studies.

In summary, in this cross-sectional study, two of the three different classification systems, SSYK and SEI gave similar results and identified groups with increased risk for respiratory symptoms and asthma, while NYK did not give conclusive results. Occupational exposure to VGDF was consistently and significantly associated with all respiratory symptoms and asthma. The increased risk for respiratory conditions associated with certain socioeconomic and occupational groups were independent of exposure to VGDF indicating that also other job-related factors than VGDF contribute to respiratory illness.
