# Supplementary material for: Job titles classified into socioeconomic and occupational groups identify subjects with increased risk for respiratory symptoms independent of occupational exposure to vapour, gas, dust, or fumes
Source: Eur Clin Respir J. 2018 May 15;5(1):1468715. doi: 10.1080/20018525.2018.1468715 (PMC5954483; doi:10.1080/20018525.2018.1468715)
Supplement: Supplemental_files.zip [file ZECR_A_1468715_SM1929.zip › Supplemental files/Online table 1_rev.docx]

|  | **Online table 1.1.** Risk for respiratory symptoms and asthma by socioeconomic status (SES) and occupational groups (NYK and SSYK) analysed by multivariable logistic regression and expressed as odds ratios (OR) and 95% confidence intervals (95% CI) stratified by working years of the longest held job in quartiles. All analyses were adjusted for sex, age, family history of asthma and smoking habits. Significant results in bold. | | | | | | | | | | | | | | | | | | | | | | | | | |
| --- | --- | --- | --- | --- | --- | --- | --- | --- | --- | --- | --- | --- | --- | --- | --- | --- | --- | --- | --- | --- | --- | --- | --- | --- | --- | --- |
|  | | Productive cough | | | | | | | | | | | |  | Recurrent wheeze | | | | | | | | | | | |
|  | | 1^st^ Q 0-14  working years | | | 2^nd^ Q 15-20  working years | | | 3^rd^ Q 21-30  working years | | | 4^th^ Q 31-60  working years | | |  | 1^st^ Q 0-14  working years | | | 2^nd^ Q 15-20  working years | | | 3^rd^ Q 21-30  working years | | | 4^th^ Q 31-60  working years | | |
|  |  | OR | 95% CI | | OR | 95% CI | | OR | 95% CI | | OR | 95% CI | |  | OR | 95% CI | | OR | 95% CI | | OR | 95% CI | | OR | 95% CI | |
| SES | |  |  |  |  |  |  |  |  |  |  |  |  |  |  |  |  |  |  |  |  |  |  |  |  |  |
| Manual workers industry | | **2.12** | **1.02** | **4.41** | 2.29 | 0.88 | 5.95 | 1.56 | 0.71 | 3.41 | 2.13 | 0.95 | 4.79 |  | 1.46 | 0.83 | 2.57 | 1.44 | 0.76 | 2.75 | **2.89** | **1.35** | **6.17** | 1.33 | 0.67 | 2.63 |
| Manual workers service | | 1.64 | 0.82 | 3.30 | **2.75** | **1.07** | **7.05** | 1.83 | 0.85 | 3.96 | 1.62 | 0.70 | 3.76 |  | 1.35 | 0.80 | 2.28 | 1.37 | 0.73 | 2.58 | **2.40** | **1.12** | **5.14** | 1.20 | 0.59 | 2.43 |
| Non-manual employees L | | 1.29 | 0.59 | 2.79 | 2.15 | 0.79 | 5.84 | 1.97 | 0.88 | 4.42 | 2.06 | 0.89 | 4.80 |  | 1.21 | 0.67 | 2.17 | 1.33 | 0.67 | 2.64 | 2.02 | 0.90 | 4.51 | 0.73 | 0.34 | 1.55 |
| Non-manual employees I | | 1.12 | 0.54 | 2.32 | 2.25 | 0.85 | 5.94 | 2.00 | 0.91 | 4.41 | 1.27 | 0.54 | 2.97 |  | 0.95 | 0.55 | 1.64 | 1.00 | 0.51 | 1.96 | **2.33** | **1.07** | **5.07** | 0.73 | 0.35 | 1.53 |
| Professionals and exec | | 1 |  |  | 1 |  |  | 1 |  |  | 1 |  |  |  | 1 |  |  | 1 |  |  | 1 |  |  | 1 |  |  |
| Self-employed non-prof | | 1.46 | 0.37 | 5.71 | 1.44 | 0.33 | 6.34 | 1.07 | 0.31 | 3.74 | 1.35 | 0.43 | 4.23 |  | 0.23 | 0.03 | 1.83 | 1.94 | 0.75 | 5.02 | 1.08 | 0.31 | 3.76 | 1.39 | 0.53 | 3.63 |
| NYK | |  |  |  |  |  |  |  |  |  |  |  |  |  |  |  |  |  |  |  |  |  |  |  |  |  |
| Science | | 0.74 | 0.40 | 1.35 | 0.91 | 0.52 | 1.61 | 1.16 | 0.73 | 1.87 | 0.66 | 0.40 | 1.09 |  | 0.72 | 0.47 | 1.13 | 0.71 | 0.43 | 1.18 | 1.25 | 0.79 | 1.98 | 0.94 | 0.53 | 1.66 |
| Health | | 1.50 | 0.92 | 2.44 | 0.89 | 0.55 | 1.45 | 0.75 | 0.47 | 1.18 | 0.49 | 0.28 | 0.87 |  | 1.04 | 0.71 | 1.53 | 1.03 | 0.69 | 1.53 | 0.98 | 0.64 | 1.50 | 1.59 | 0.95 | 2.65 |
| Administration | | 1 |  |  | 1 |  |  | 1 |  |  | 1 |  |  |  | 1 |  |  | 1 |  |  | 1 |  |  | 1 |  |  |
| Agriculture | | 0.94 | 0.31 | 2.87 | 1.24 | 0.52 | 2.95 | 0.94 | 0.40 | 2.24 | 0.85 | 0.39 | 1.87 |  | 0.33 | 0.10 | 1.11 | 1.27 | 0.60 | 2.71 | 1.26 | 0.58 | 2.76 | 1.72 | 0.78 | 3.80 |
| Mining | | 0.61 | 0.08 | 4.92 | 1.91 | 0.70 | 5.16 | 1.14 | 0.36 | 3.59 | 0.92 | 0.38 | 2.22 |  | 1.12 | 0.31 | 4.09 | 0.97 | 0.34 | 2.76 | 1.97 | 0.73 | 5.29 | 1.52 | 0.61 | 3.80 |
| Transportation | | 0.78 | 0.31 | 1.93 | 1.27 | 0.62 | 2.59 | 1.04 | 0.54 | 1.98 | 0.62 | 0.31 | 1.23 |  | 0.63 | 0.31 | 1.29 | 1.23 | 0.66 | 2.29 | 1.71 | 0.97 | 3.02 | 1.37 | 0.70 | 2.68 |
| Manufacturing | | 1.44 | 0.85 | 2.45 | 0.88 | 0.53 | 1.45 | 0.90 | 0.55 | 1.46 | 1.17 | 0.75 | 1.83 |  | 1.14 | 0.75 | 1.74 | 1.08 | 0.71 | 1.64 | **1.66** | **1.07** | **2.56** | **2.08** | **1.26** | **3.42** |
| Service | | **1.88** | **1.08** | **3.25** | 0.82 | 0.45 | 1.49 | 0.84 | 0.48 | 1.47 | 0.99 | 0.50 | 1.93 |  | 0.90 | 0.55 | 1.46 | 1.09 | 0.67 | 1.75 | 0.82 | 0.47 | 1.43 | 1.64 | 0.81 | 3.32 |
| SSYK | |  |  |  |  |  |  |  |  |  |  |  |  |  |  |  |  |  |  |  |  |  |  |  |  |  |
| Managers | | 1 |  |  | 1 |  |  | 1 |  |  | 1 |  |  |  | 1 |  |  | 1 |  |  | 1 |  |  | 1 |  |  |
| Occupations req. advanced education | | 2.21 | 0.51 | 9.67 | b |  |  | 2.10 | 0.48 | 9.20 | 0.72 | 0.20 | 2.63 |  | 1.17 | 0.44 | 3.14 | 5.24 | 0.68 | 40.2 | 1.31 | 0.38 | 4.51 | 0.71 | 0.19 | 2.62 |
| Occupations req. higher education | | 1.78 | 0.38 | 8.36 | b |  |  | 2.75 | 0.61 | 12.3 | 0.65 | 0.17 | 2.51 |  | 1.18 | 0.42 | 3.32 | 3.41 | 0.43 | 27.0 | 1.68 | 0.47 | 5.99 | 0.64 | 0.16 | 2.49 |
| Administration | | 2.47 | 0.54 | 11.3 | b |  |  | 2.36 | 0.53 | 10.5 | 1.33 | 0.37 | 4.76 |  | 1.45 | 0.52 | 4.07 | 5.70 | 0.74 | 44.1 | 1.54 | 0.44 | 5.37 | 0.62 | 0.17 | 2.32 |
| Service | | 3.23 | 0.75 | 13.8 | b |  |  | 1.54 | 0.35 | 6.73 | 1.14 | 0.32 | 4.10 |  | 2.07 | 0.78 | 5.44 | 5.48 | 0.72 | 41.4 | 1.19 | 0.35 | 4.07 | 1.22 | 0.34 | 4.41 |
| Agriculture | | 3.71 | 0.70 | 19.7 | b |  |  | 1.58 | 0.30 | 8.35 | 0.99 | 0.24 | 4.03 |  | 0.53 | 0.12 | 2.41 | 6.69 | 0.80 | 55.7 | 1.93 | 0.49 | 7.54 | 1.38 | 0.34 | 5.54 |
| Building | | 3.71 | 0.84 | 16.3 | b |  |  | 1.58 | 0.36 | 7.05 | 1.05 | 0.30 | 3.68 |  | 1.79 | 0.66 | 4.85 | 6.17 | 0.81 | 47.1 | 2.37 | 0.69 | 8.10 | 1.10 | 0.31 | 3.90 |
| Manufacturing | | 2.57 | 0.55 | 12.0 | b |  |  | 1.81 | 0.40 | 8.05 | 1.28 | 0.36 | 4.47 |  | 1.48 | 0.52 | 4.21 | 6.94 | 0.91 | 53.2 | 2.39 | 0.70 | 8.22 | 1.58 | 0.45 | 5.58 |
| Elementary | | **5.23** | **1.17** | **23.3** | b |  |  | 2.77 | 0.60 | 12.7 | 0.61 | 0.09 | 4.06 |  | 1.85 | 0.65 | 5.26 | **8.40** | **1.07** | **65.8** | 1.36 | 0.37 | 5.01 | 1.09 | 0.21 | 5.70 |
|  | b cannot be calculated due to few cases | | | | | | | | | | | | | | | | | | | | | | | | | |

| **Online table 1.2.** Risk for respiratory symptoms and asthma by socioeconomic status (SES) and occupational groups (NYK and SSYK) analysed by multivariable logistic regression and expressed as odds ratios (OR) and 95% confidence intervals (95% CI) stratified by working years of the longest held job in quartiles. All analyses were adjusted for sex, age, family history of asthma and smoking habits. Significant results in bold, borderline significant results in bold italic. | | | | | | | | | | | | |
| --- | --- | --- | --- | --- | --- | --- | --- | --- | --- | --- | --- | --- |
|  | Current asthma | | | | | | | | | | | |
|  | 1^st^ Q 0-14  working years | | | 2^nd^ Q 15-20  working years | | | 3^rd^ Q 21-30  working years | | | 4^th^ Q 31-60  working years | | |
|  | OR | 95% CI | | OR | 95% CI | | OR | 95% CI | | OR | 95% CI | |
| SES |  |  |  |  |  |  |  |  |  |  |  |  |
| Manual workers industry | 1.08 | 0.59 | 1.99 | 0.97 | 0.54 | 1.76 | 2.15 | 0.89 | 5.18 | 0.89 | 0.42 | 1.92 |
| Manual workers service | 1.14 | 0.66 | 1.97 | 0.91 | 0.51 | 1.63 | **2.71** | **1.14** | **6.47** | 0.95 | 0.44 | 2.07 |
| Non-manual employees L | 0.92 | 0.50 | 1.72 | 0.79 | 0.42 | 1.51 | 1.95 | 0.78 | 4.88 | 0.49 | 0.21 | 1.13 |
| Non-manual employees I | 0.95 | 0.54 | 1.66 | 0.71 | 0.38 | 1.31 | **2.55** | **1.05** | **6.18** | 0.82 | 0.38 | 1.80 |
| Professionals and exec | 1 |  |  | 1 |  |  | 1 |  |  | 1 |  |  |
| Self-employed non-prof | 0.87 | 0.24 | 3.21 | 1.03 | 0.38 | 2.81 | 1.01 | 0.24 | 4.20 | 0.66 | 0.19 | 2.29 |
| NYK |  |  |  |  |  |  |  |  |  |  |  |  |
| Science | 0.86 | 0.54 | 1.35 | 1.16 | 0.71 | 1.90 | 1.34 | 0.80 | 2.24 | 0.93 | 0.50 | 1.72 |
| Health | 1.03 | 0.68 | 1.55 | 1.11 | 0.72 | 1.70 | 1.20 | 0.75 | 1.93 | 1.60 | 0.92 | 2.76 |
| Administration | 1 |  |  | 1 |  |  | 1 |  |  | 1 |  |  |
| Agriculture | 0.45 | 0.13 | 1.52 | 1.06 | 0.45 | 2.50 | 1.24 | 0.52 | 2.99 | 2.19 | 0.95 | 5.09 |
| Mining | b |  |  | 0.25 | 0.03 | 1.89 | 0.87 | 0.19 | 3.93 | 1.15 | 0.37 | 3.60 |
| Transportation | 0.40 | 0.15 | 1.03 | 1.26 | 0.63 | 2.54 | **1.94** | **1.02** | **3.71** | 0.88 | 0.38 | 2.08 |
| Manufacturing | 1.03 | 0.64 | 1.65 | 1.08 | 0.68 | 1.70 | 1.36 | 0.81 | 2.29 | 1.38 | 0.77 | 2.49 |
| Service | 0.78 | 0.44 | 1.36 | 1.24 | 0.74 | 2.10 | 1.27 | 0.71 | 2.27 | 1.70 | 0.79 | 3.65 |
| SSYK |  |  |  |  |  |  |  |  |  |  |  |  |
| Managers | 1 |  |  | 1 |  |  | 1 |  |  | 1 |  |  |
| Occupations req. advanced education | 0.92 | 0.34 | 2.48 | b |  |  | 4.01 | 0.53 | 30.4 | 1.04 | 0.22 | 4.85 |
| Occupations req. higher education | 1.07 | 0.38 | 2.99 | b |  |  | 4.77 | 0.61 | 37.1 | 0.75 | 0.15 | 3.80 |
| Administration | 0.67 | 0.23 | 1.99 | b |  |  | 4.62 | 0.60 | 35.4 | 0.65 | 0.14 | 3.13 |
| Service | 1.37 | 0.52 | 3.62 | b |  |  | 3.56 | 0.47 | 26.9 | 1.17 | 0.25 | 5.44 |
| Agriculture | 0.74 | 0.18 | 2.98 | b |  |  | 3.87 | 0.45 | 33.0 | 2.25 | 0.44 | 11.36 |
| Building | 1.42 | 0.51 | 3.93 | b |  |  | 4.10 | 0.54 | 31.3 | 1.00 | 0.22 | 4.60 |
| Manufacturing | 0.55 | 0.17 | 1.78 | b |  |  | 4.09 | 0.53 | 31.6 | 1.41 | 0.31 | 6.45 |
| Elementary | 0.96 | 0.32 | 2.87 | b |  |  | 4.42 | 0.55 | 35.2 | 0.37 | 0.03 | 4.54 |
| b cannot be calculated due to few cases | | | | | | | | | | | | |
