# Supplementary material for: Job titles classified into socioeconomic and occupational groups identify subjects with increased risk for respiratory symptoms independent of occupational exposure to vapour, gas, dust, or fumes
Source: Eur Clin Respir J. 2018 May 15;5(1):1468715. doi: 10.1080/20018525.2018.1468715 (PMC5954483; doi:10.1080/20018525.2018.1468715)
Supplement: Supplemental_files.zip [file ZECR_A_1468715_SM1929.zip › Supplemental files/Online Table 2.docx]

| **Online Table 2**. Risk for respiratory symptoms and conditions by socioeconomic status (SES), occupational groups (NYK and SSYK) and by self-reported occupational exposure to VGDF analysed by multivariable logistic regression analyses and expressed as odds ratios (OR) and 95% confidence intervals (95% CI). Subjects with asthma onset before the age of 18 years were excluded from the population. All analyses were adjusted for sex, age, family history of asthma and smoking habits. Significant results in bold. | | | | | | | | | | | | | | | | | | | | | | | | |
| --- | --- | --- | --- | --- | --- | --- | --- | --- | --- | --- | --- | --- | --- | --- | --- | --- | --- | --- | --- | --- | --- | --- | --- | --- |
|  | Productive cough | | | Recurrent wheeze | | | Asthmatic wheeze | | | Allergic rhinoconjunctivitis | | | Rhinitis | | | Current asthma | | | Allergic asthma | | | Non-allergic asthma | | |
|  | OR | 95% CI | | OR | 95% CI | | OR | 95% CI | | OR | 95% CI | | OR | 95% CI | | OR | 95% CI | | OR | 95% CI | | OR | 95% CI | |
| SES |  |  |  |  |  |  |  |  |  |  |  |  |  |  |  |  |  |  |  |  |  |  |  |  |
| Manual workers industry | **2.25** | **1.26** | **4.01** | 1.30 | 0.89 | 1.89 | 1.78 | 0.92 | 3.45 | 1.02 | 0.74 | 1.41 | 1.29 | 0.93 | 1.78 | 1.37 | 0.78 | 2.40 | 1.84 | 0.75 | 4.52 | 1.06 | 0.53 | 2.12 |
| Manual workers service | 1.78 | 0.98 | 3.13 | 1.04 | 0.71 | 1.50 | 1.61 | 0.84 | 3.08 | 0.98 | 0.71 | 1.33 | 1.00 | 0.73 | 1.38 | 1.17 | 0.65 | 1.92 | 1.79 | 0.76 | 4.22 | 0.74 | 0.37 | 1.48 |
| Non-manual employees L | 1.79 | 0.98 | 3.27 | 1.16 | 0.78 | 1.72 | 1.53 | 0.77 | 3.03 | 1.08 | 0.77 | 1.51 | 1.00 | 0.71 | 1.42 | 0.88 | 0.49 | 1.59 | 1.56 | 0.46 | 2.92 | 0.75 | 0.36 | 1.56 |
| Non-manual employees I | 1.54 | 0.85 | 2.81 | 0.92 | 0.63 | 1.36 | 1.27 | 0.65 | 2.51 | 1.14 | 0.83 | 1.57 | 0.86 | 0.62 | 1.20 | 0.87 | 0.49 | 1.54 | 1.68 | 0.70 | 4.06 | **0.44** | **0.21** | **0.96** |
| Professionals and exec | 1 |  |  | 1 |  |  | 1 |  |  | 1 |  |  | 1 |  |  | 1 |  |  | 1 |  |  | 1 |  |  |
| Self-employed non-prof | 1.58 | 0.67 | 7.70 | 1.10 | 0.60 | 2.01 | 2.23 | 0.90 | 5.48 | 0.75 | 0.43 | 1.32 | 0.79 | 0.45 | 1.41 | 0.96 | 0.38 | 2.41 | 0.84 | 0.16 | 4.25 | 0.98 | 0.33 | 2.93 |
| NYK |  |  |  |  |  |  |  |  |  |  |  |  |  |  |  |  |  |  |  |  |  |  |  |  |
| Science | 0.81 | 0.56 | 1.19 | 0.90 | 0.68 | 1.20 | 0.79 | 0.50 | 1.22 | 1.05 | 0.83 | 1.33 | 1.11 | 0.87 | 1.42 | 0.77 | 0.50 | 1.20 | 0.78 | 0.44 | 1.37 | 0.77 | 0.39 | 1.50 |
| Healthcare | 0.78 | 0.55 | 1.09 | 0.98 | 0.76 | 1.26 | 0.94 | 0.65 | 1.37 | 0.82 | 0.66 | 1.02 | 0.96 | 0.76 | 1.20 | 1.19 | 0.83 | 1.70 | 1.04 | 0.66 | 1.65 | 1.37 | 0.80 | 2.34 |
| Administration | 1 |  |  | 1 |  |  | 1 |  |  | 1 |  |  | 1 |  |  | 1 |  |  | 1 |  |  | 1 |  |  |
| Agriculture | 1.67 | 0.97 | 2.85 | 1.14 | 0.70 | 1.84 | 1.32 | 0.68 | 2.60 | 0.99 | 0.64 | 1.53 | 1.12 | 0.72 | 1.74 | 1.49 | 0.78 | 2.87 | 1.11 | 0.42 | 2.97 | 1.88 | 0.81 | 4.35 |
| Mining | 1.52 | 0.73 | 3.18 | 1.87 | 1.04 | 3.36 | 1.27 | 0.51 | 3.16 | 1.00 | 0.53 | 1.90 | 1.14 | 0.61 | 2.13 | 0.41 | 0.10 | 1.77 | 0.48 | 0.06 | 3.66 | 0.39 | 0.05 | 3.00 |
| Transportation | 1.05 | 0.64 | 1.71 | 1.05 | 0.72 | 1.54 | 1.17 | 0.67 | 2.04 | 1.06 | 0.75 | 1.50 | 1.12 | 0.79 | 1.58 | 0.64 | 0.31 | 1.28 | 0.79 | 0.32 | 1.95 | 0.52 | 0.18 | 1.53 |
| Manufacturing | 1.17 | 0.84 | 1.63 | 1.18 | 0.91 | 1.54 | 1.09 | 0.74 | 1.62 | 0.98 | 0.77 | 1.24 | 1.27 | 1.00 | 1.61 | 1.34 | 0.91 | 1.98 | 1.35 | 0.80 | 2.30 | 1.33 | 0.76 | 2.31 |
| Service | 0.95 | 0.63 | 1.43 | 0.82 | 0.59 | 1.14 | 0.79 | 0.48 | 1.29 | 0.84 | 0.63 | 1.11 | 0.94 | 0.70 | 1.25 | 1.27 | 0.82 | 1.98 | 0.74 | 0.38 | 1.41 | **2.07** | **1.15** | **3.72** |
| SSYK |  |  |  |  |  |  |  |  |  |  |  |  |  |  |  |  |  |  |  |  |  |  |  |  |
| Managers | 1 |  |  | 1 |  |  | 1 |  |  | 1 |  |  | 1 |  |  | 1 |  |  | 1 |  |  | 1 |  |  |
| Occupations req. advanced education | 1.85 | 0.73 | 5.30 | 1.24 | 0.66 | 2.35 | 1.11 | 0.48 | 2.56 | 1.14 | 0.67 | 1.94 | 0.81 | 0.48 | 1.35 | 0.98 | 0.40 | 2.41 | 1.01 | 0.30 | 3.49 | 0.93 | 0.27 | 3.27 |
| Occupations req. higher education | 2.15 | 0.73 | 6.35 | 1.04 | 0.53 | 2.03 | 0.82 | 0.50 | 1.35 | 1.01 | 0.58 | 1.76 | 0.72 | 0.42 | 1.26 | 1.02 | 0.39 | 2.66 | 1.03 | 0.28 | 3.82 | 1.01 | 0.27 | 3.81 |
| Administration | 2.56 | 0.89 | 7.36 | 1.34 | 0.70 | 2.57 | 1.07 | 0.62 | 1.86 | 1.19 | 0.69 | 2.04 | 0.88 | 0.52 | 1.50 | 0.87 | 0.34 | 2.20 | 0.89 | 0.25 | 3.16 | 0.87 | 0.24 | 3.15 |
| Service | 2.25 | 0.80 | 6.38 | 1.33 | 0.71 | 2.50 | 1.00 | 0.60 | 1.68 | 0.96 | 0.57 | 1.62 | 0.88 | 0.53 | 1.46 | 1.27 | 0.52 | 3.07 | 1.34 | 0.40 | 4.50 | 1.15 | 0.34 | 3.90 |
| Agriculture | **3.46** | **1.12** | **10.65** | 1.51 | 0.72 | 3.17 | 1.13 | 0.72 | 1.79 | 1.05 | 0.56 | 2.00 | 1.01 | 0.54 | 1.87 | 1.67 | 0.60 | 4.67 | 1.28 | 0.29 | 5.61 | 1.97 | 0.50 | 7.77 |
| Building | 2.54 | 0.89 | 7.23 | 1.43 | 0.76 | 2.70 | 1.27 | 0.63 | 2.56 | 1.02 | 0.60 | 1.74 | 1.12 | 0.67 | 1.88 | 1.51 | 0.61 | 3.72 | 1.39 | 0.39 | 4.88 | 1.56 | 0.46 | 5.37 |
| Manufacturing | **3.08** | **1.08** | **8.79** | 1.76 | 0.93 | 3.34 | 1.08 | 0.66 | 1.77 | 1.00 | 0.54 | 1.74 | 0.86 | 0.51 | 1.47 | 1.23 | 0.49 | 3.10 | 1.33 | 0.37 | 4.76 | 1.13 | 0.32 | 4.02 |
| Elementary | **3.28** | **1.11** | **9.71** | 1.16 | 0.58 | 2.33 | 1.39 | 0.84 | 2.30 | 0.97 | 0.37 | 1.76 | 0.88 | 0.67 | 1.56 | 1.18 | 0.45 | 3.12 | 0.83 | 0.21 | 3.29 | 1.60 | 0.43 | 5.98 |
| Occupational exposure |  |  |  |  |  |  |  |  |  |  |  |  |  |  |  |  |  |  |  |  |  |  |  |  |
| No VGDF | 1 |  |  | 1 |  |  | 1 |  |  | 1 |  |  | 1 |  |  | 1 |  |  | 1 |  |  | 1 |  |  |
| VGDF | **2.39** | **1.91** | **2.97** | **2.01** | **1.69** | **2.39** | **2.47** | **1.90** | **3.20** | **1.40** | **1.20** | **1.65** | **1.87** | **1.59** | **2.19** | **2.08** | **1.61** | **2.68** | **2.33** | **1.65** | **3.29** | **1.70** | **1.19** | **2.42** |
|  | | | | | | | | | | | | | | | | | | | | | | | | |
