# Supplementary material for: Job titles classified into socioeconomic and occupational groups identify subjects with increased risk for respiratory symptoms independent of occupational exposure to vapour, gas, dust, or fumes
Source: Eur Clin Respir J. 2018 May 15;5(1):1468715. doi: 10.1080/20018525.2018.1468715 (PMC5954483; doi:10.1080/20018525.2018.1468715)
Supplement: Supplemental_files.zip [file ZECR_A_1468715_SM1929.zip › Supplemental files/Online Table 3.docx]

| **Online Table 3.** Risk for respiratory symptoms and asthma analysed by multivariable logistic regression and expressed as odds ratios (OR) and 95% confidence intervals (95% CI). Significant results in bold. | | | | | | | | | | | | | | | | | | | | | | | | |
| --- | --- | --- | --- | --- | --- | --- | --- | --- | --- | --- | --- | --- | --- | --- | --- | --- | --- | --- | --- | --- | --- | --- | --- | --- |
|  | Productive cough | | | Recurrent wheeze | | | Asthmatic wheeze | | | Allergic rhino-conjunctivitis | | | Rhinitis | | | Current asthma | | | Allergic asthma | | | Non-allergic asthma | | |
|  | OR | 95% CI | | OR | 95% CI | | OR | 95% CI | | OR | 95% CI | | OR | 95% CI | | OR | 95% CI | | OR | 95% CI | | OR | 95% CI | |
| Age (continuous) | **1.02** | **1.01** | **1.03** | 1.00 | 0.99 | 1.01 | 1.01 | 1.00 | 1.02 | **0.97** | **0.97** | **0.98** | 0.99 | 0.98 | 1.00 | 1.00 | 0.99 | 1.01 | 0.98 | 0.97 | 1.00 | 1.01 | 1.00 | 1.03 |
| Male | 1.10 | 0.92 | 1.33 | 0.98 | 0.85 | 1.13 | 0.94 | 0.77 | 1.16 | **0.81** | **0.73** | **0.91** | **0.81** | **0.71** | **0.92** | **0.80** | **0.66** | **0.96** | **0.35** | **0.28** | **045** | **0.50** | **0.37** | **0.66** |
| Female | 1 |  |  | 1 |  |  | 1 |  |  | 1 |  |  | 1 |  |  | 1 |  |  | 1 |  |  | 1 |  |  |
| No fam history of asthma | 1 |  |  | 1 |  |  | 1 |  |  | 1 |  |  | 1 |  |  | 1 |  |  | 1 |  |  | 1 |  |  |
| Family history of asthma | **1.98** | **1.62** | **2.42** | **2.21** | **1.89** | **2.58** | **2.95** | **2.38** | **3.66** | **2.07** | **1.87** | **2.30** | **1.74** | **1.50** | **2.02** | **3.35** | **2.76** | **4.07** | **3.50** | **2.75** | **4.44** | **2.36** | **1.77** | **3.15** |
| Non-smoker | 1 |  |  | 1 |  |  | 1 |  |  | 1 |  |  | 1 |  |  | 1 |  |  | 1 |  |  | 1 |  |  |
| Ex-smoker | **1.49** | **1.18** | **1.88** | **1.49** | **1.25** | **1.78** | **1.76** | **1.36** | **2.67** | 0.86 | 0.74 | 1.01 | **1.20** | **1.02** | **1.41** | **1.40** | **1.12** | **1.76** | 1.22 | 0.92 | 1.62 | **1.72** | **1.25** | **2.38** |
| Current smoker | **2.73** | **2.19** | **3.41** | **2.93** | **2.47** | **3.48** | **2.22** | **1.71** | **2.87** | 0.76 | 0.63 | 0.90 | **1.57** | **1.33** | **1.86** | 1.17 | 0.90 | 1.50 | 0.61 | 0.79 | 1.49 | 1.33 | 0.92 | 1.94 |
